# Identification of Novel Mutations in *Arabidopsis thaliana* DOF 4.2 Coding Gene

**DOI:** 10.34172/apb.2021.064

**Published:** 2020-05-09

**Authors:** Omid Jamshidi Kandjani, Mahdieh Rahbar-Shahrouziasl, Ali Akbar Alizadeh, Maryam Hamzeh-Mivehroud, Siavoush Dastmalchi

**Affiliations:** ^1^Biotechnology Research Center, Tabriz University of Medical Sciences, Tabriz, Iran.; ^2^School of Pharmacy, Tabriz University of Medical Sciences, Tabriz, Iran.; ^3^Pharmaceutical Analysis Research Center, Tabriz University of Medical Sciences, Tabriz, Iran.

**Keywords:** DOF 4.2-ZF, Gene cloning, Protein expression, Homology modeling, Molecular dynamics simulation

## Abstract

***Purpose:*** DOF (DNA-binding with One Finger) proteins are plant-specific transcription factors which mediate numerous biological processes. The purpose of the current study is to report new naturally occurring mutations in the gene encoding for one of the members of DOF proteins named DOF 4.2.

***Methods:*** The expression of zinc finger domain of DOF 4.2 (DOF 4.2-ZF) was investigated by first synthesis of cDNA library using different parts of *Arabidopsis thaliana* plant. Then the coding sequence for zinc finger domain of DOF 4.2 protein was prepared using nested PCR experiment and cloned into pGEX-6P-1 expression vector. Finally, the prepared construct was used for protein expression. Furthermore, molecular dynamics (MD) simulation was carried out to predict DNA binding affinity of DOF 4.2-ZF using AMBER package.

***Results:*** For the first time a new variant of DOF 4.2-ZF protein with three mutations was detected. One of the mutations is silent while the other two mutations lead to amino acid replacement (S18G) as well as introduction of a stop codon ultimately resulting in a truncated protein production. In order to investigate whether the truncated form is able to recognize DNA binding motif, MD simulations were carried out and the results showed that the chance of binding of DOF 4.2-ZF protein to cognate DNA in its truncated form is very low.

***Conclusion:*** The findings demonstrated that the observed mutations adversely affect the DNA binding ability of the truncated form of DOF4.2 if it is expressed in the mutant variant of *A. thaliana* used in this study.

## Introduction

Different classes of transcription factors have been evolved to specifically contribute to the regulation of plant gene expression as well as mediating plant-specific signals.^1^ DOF (DNA-binding with One Finger) proteins are a family of plant-specific transcription factors which contribute to numerous biological processes upon binding to DNA through a highly conserved DNA binding domain. This family of transcription factors has been discovered in both monocots and dicots as well. Genome analysis has revealed that *Arabidopsis*genome has 37 putative DOF genes playing essential roles in plant gene expression.^2^ DOF proteins are typically composed of a conserved DNA-binding domain and a transcriptional regulation domain organized at N-terminal and C-terminal ends, respectively, which have been linked to each other by conjunctive sequences.^3^ The N-terminal DNA-binding domain can recognize various proteins and especially DNA sequences harboring an AAAG core motif.^4,5^ Since the discovery of first DOF protein (i.e., ZmDOF1) as a regulator of light response in maize, many attempts have been dedicated to understand DOF proteins versatile roles in biological processes in different plant species.^3^ It was later observed that two other maize DOF proteins, namely ZmDOF2 and ZmPBF, contribute in regulation of light response and seed germination, respectively.^6,7^ Further studies on DOF family proteins in barely revealed that HvPBF, HvSAD, HvDOF17, and HvDOF19 DOF transcription proteins are involved in regulating the expression of different aleurone hydrolase genes,^8,9^ leading to seed germination. In *Arabidopsis*, DOF proteins act as the mediators of light responses,^10^ flowering,^11^ plant growth,^12,13^ hormone response,^14,15^ cell cycle regulation,^16^ secondary metabolism,^17^ cambium formation and vascular tissue development,^18^ as well as seed germination.^15,19^ In addition, DOF transcription factors control ammonium assimilation,^20^ carbohydrate metabolism,^21^ and fatty acid synthesis.^22^ DOF transcription factors exert their biological activities through binding either directly or indirectly to the promoters of target genes to regulate downstream signaling pathways. All these facts signify the core regulatory effect of these transcription factors in biological processes determining the amount and quality of plant metabolites and biomass. Several ground-breaking studies have demonstrated that, in contrary to most structural genes, transcription factors are involved in controlling multiple pathways.^23^ Therefore, it’s of great importance to explore the function of transcription factors in order to provide understandings applicable for the development of transcription factor-based technology to improve the quality and the efficiency of the plant materials.^23-25^ As *Arabidopsis*genome sequence has been completed, it is possible to identify putative genes for transcription factors on a genome-wide scale.^2^ In the current study we aimed to isolate the gene encoding for DOF 4.2-ZF protein from a native variant of *Arabidopsis thaliana*grown in greenhouse. However, it was noticed that the used variant carries a truncated form of DOF 4.2-ZF DNA sequence not reported previously. Translation of the mutated DOF 4.2-ZF gene revealed three mutations which were missense, silent and nonsense, resulting in a truncated form of protein. Therefore, in the current study, molecular dynamics simulations were performed to explore the interaction of this truncated form of DOF 4.2-ZF to DNA and compare the results to that of wild type.

## Materials and Methods

### Cloning of DOF4.2-ZF into pGEX-6P-1 vector

**The complete body of***A. thaliana* was disrupted for RNA extraction. In order to prevent degradation of RNA, all equipments were double autoclaved to inactivate RNase. QIAGEN RNeasy plant minikit was employed for RNA extraction as described in the manufacturer’s instruction. Briefly, the plant tissue was grounded thoroughly in liquid nitrogen and the obtained powder was decanted into RNase-free liquid-nitrogen–cooled 2 mL microcentrifuge tube. Subsequently, RLT buffer was added to 100 mg of tissue powder and after complete vortexing, the clear lysate was transferred to a QIAshredder spin column and centrifuged for 2 min at 13,000 *g*. Then, half volume of absolute ethanol was added to the flow-through and the mixture was transferred to an RNeasy Mini spin column and centrifuged for 15 seconds at ≥8000* g*. The process was proceeded by addition of Buffer RW1 (700 μL) to the RNeasy spin column followed by centrifugation for 15 seconds at ≥8000 *g.* The sample was further washed using 500 μL of Buffer RPE. Finally, RNA was eluted from column membrane using 40 μL RNase-free water and kept in -20°C until cDNA synthesis. The cDNA library was synthesized using the prepared mRNA from plant *A. thaliana* using RevertAid cDNA synthesis kit from Fermentas. For the synthesis of cDNA, 3 μL RNA, 1 μ Loligo-dT and 12 μL DEPC treated water were added into a 1 mL sterile tube and incubated in 70 °C for 5 minutes. Then the tube was placed on ice while 4 μL reaction buffer, 1 μL RNase inhibitor and 2 μL dNTPs were added with additional incubation at 37 °C for 5 minutes. In the next step, 1 μL reverse transcriptase enzyme was added and the mixture was incubated in 42°C for 60 minutes. Finally, the mixture was incubated in 70°C for 10 minutes. Amplification of the desired DNA sequence was carried out using the produced cDNA as the template in the nested PCR reaction as follows: at first, using outer primers, F1 (5’GAAATGAATGTGATGCCCCCACCG 3^’^) andR1 (5’ATGATTGCCTTGTTGGATCTCAGCC 3^’^) a larger sequence was amplified and the obtained PCR product was used in the second PCR reaction using inner primers F2 (5’ GAAGGATCCGAAATGAATGTGATGC 3’) and R2 (5’ TTCGGGAATTCTCATTTATCACATATTCC 3’). The product of the latter PCR reaction was electrophoresed on 1% agarose gel followed by gel purification. BamHI and EcoRI restriction enzymes were used to cut the PCR product at 37°C for 4 hours and cloned into the pGEX-6P-1 vector which was already cut with the same enzymes. Using T4 DNA ligase DOF 4.2 ZF domain coding sequence was cloned into pGEX-6P-1 at 16°C for 16 hours and the reaction mixture was transformed into *E. coli DH5α* strain. The transformed bacteria were cultured on LB-agar plates with 100 µg/mL ampicillin and the obtained single colonies were investigated for the production of DOF 4.2-ZF-pGEX-6P-1 vector. To do so, the single colonies were used to inoculate 10 mL LB medium with 100 µg/mL ampicillin and cultured for overnight at 37°C. In the next day, the bacteria culture was used to extract plasmids using plasmid extraction mini kit. Two PCR reactions were carried out on the extracted plasmid using DOF 4.2-ZF F2 and R2 specific as well as pGEX universal primers to check the validity of ligation process. Finally, the constructed vector was sequenced for final approval.

### Expression of DOF4.2 ZF protein

The constructed DOF4.2 ZF-pGEX-6P-1 vector was transformed into*E. coli BL21*and a single colony was inoculated into 10 mL LB-ampicillin medium for overnight growth 37°C. The bacterial culture was diluted 1:100 in LB-ampicillin and incubated at 37°C while shaking at 170 rpm. At OD of 0.6, IPTG was added to the culture and the culturing temperature was set to 20°C for overnight protein expression. Different samples before and after induction were taken to monitor protein expression. The next day, the bacteria were separated from medium by centrifugation at 5000 rpm for 15 minutes and suspended in lysis buffer containing Tris 50 mM pH 8, NaCl 150 mM, Triton 1%, lysozyme 0.1 mg/mL, DNAse 10 µg/mL, β-mercaptoethanol 0.1%, PMSF 1.4 mM). Using three cycles of freeze-thaw followed by sonication on ice five times for 30 seconds at 60 pulse the protein content of bacteria was released. In order to separate soluble fraction containing the protein of interest from bacterial debris, the suspension was centrifuged at 10 000 rpm for 30 minutes at 4°C. Expression of protein was evaluated using SDS-PAGE analysis.

### Model building and molecular dynamics simulation studies

**Figure 1:**
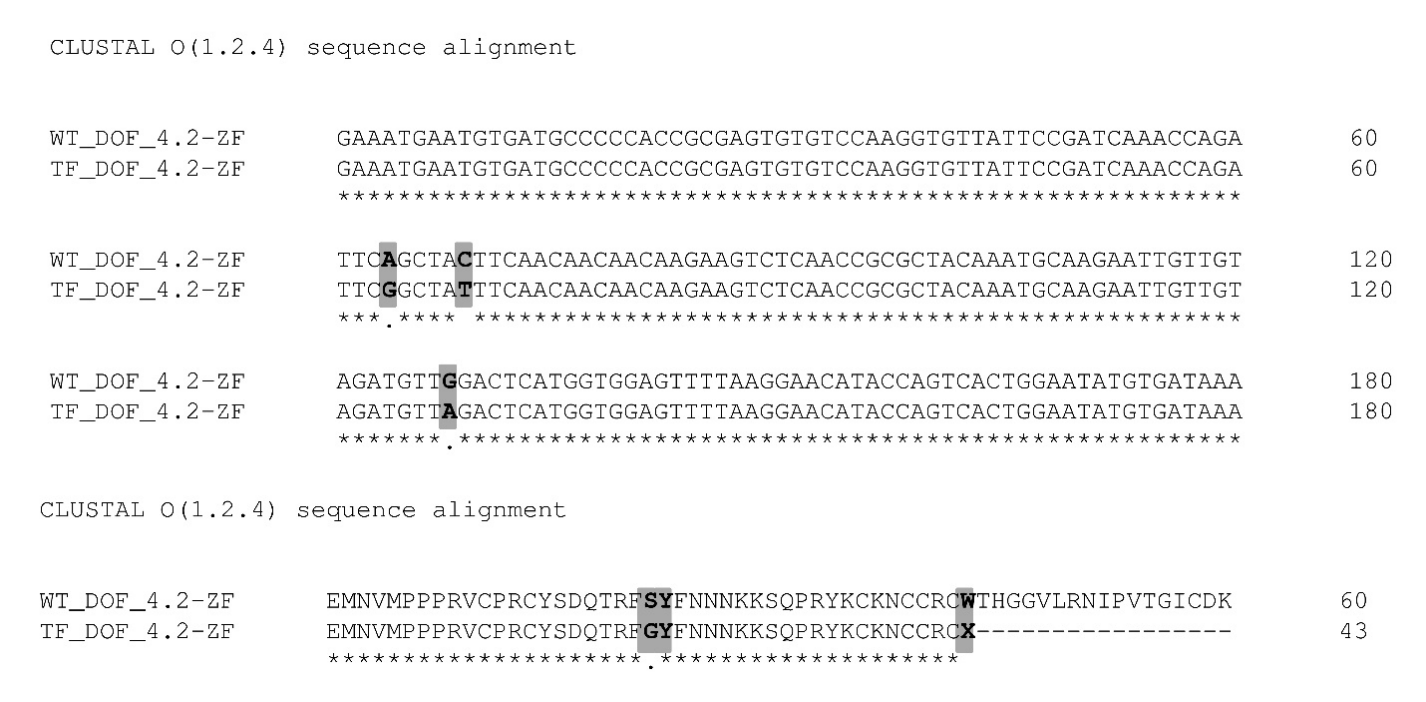
The DNA and protein sequence alignments between wild type and truncated form of DOF 4.2-ZF. The upper and lower sequence alignments refer to the DNA and protein alignment, respectively. An asterisk (*) indicates positions which have a single, fully conserved residue and a period (.) indicates conservation between groups of weakly similar properties scoring ≤ 0.5 in the Gonnet PAM 250 matrix. The position of mutations is highlighted in gray. In protein sequence alignment, the “X” character indicates end of translation in the truncated DOF-4.2-ZF sequence.

All sequences and 3D experimental structures were retrieved from UniProt and RCSB databases, respectively. The amino acid sequence for the truncated form of DOF 4.2-ZF protein used in the current study was shown in Figure 1. The 3D model for truncated form of DOF 4.2-ZF protein was derived from the previously proposed model for the wild type protein.^26^ The generated model was subjected to molecular dynamics (MD) studies using AMBER suite of programs (version 14) operating on a Linux-based (Centus 6.8) GPU workstation. Initially, the parameter files for the complex of DNA and DOF 4.2 ZF domain were generated using leap module implemented in AMBER program. Then the total charge of the system was neutralized by adding the correct numbers of counter ions (Na^+^ or Cl^−^), followed by solvating the system with TIP3P water molecules allowing a buffering distance of 12 Å in all directions in the simulation box. The generated system was equilibrated by applying a brief energy minimization using 500 steps of steepest descent and 500 steps of conjugate gradient minimizations, 50 ps heating step, where the temperature was increased from 0 to 300°K, 50 ps density equilibration, and constant pressure equilibration for 500 ps at 300°K. Using the SHAKE algorithm the bond lengths involving hydrogen atoms were constrained. By employing the particle mesh Ewald (PME) method, the final MD simulation was carried out for 20 ns with a time step of 2 fs. The trajectories were obtained by writing out the coordinates every 10 ps. The RMSD values were calculated using CPPTRAJ module of Amber Tools.

## Results

### Cloning of DOF4.2ZF domain sequence into pGEX-6P-1 vector

**Figure 2:**
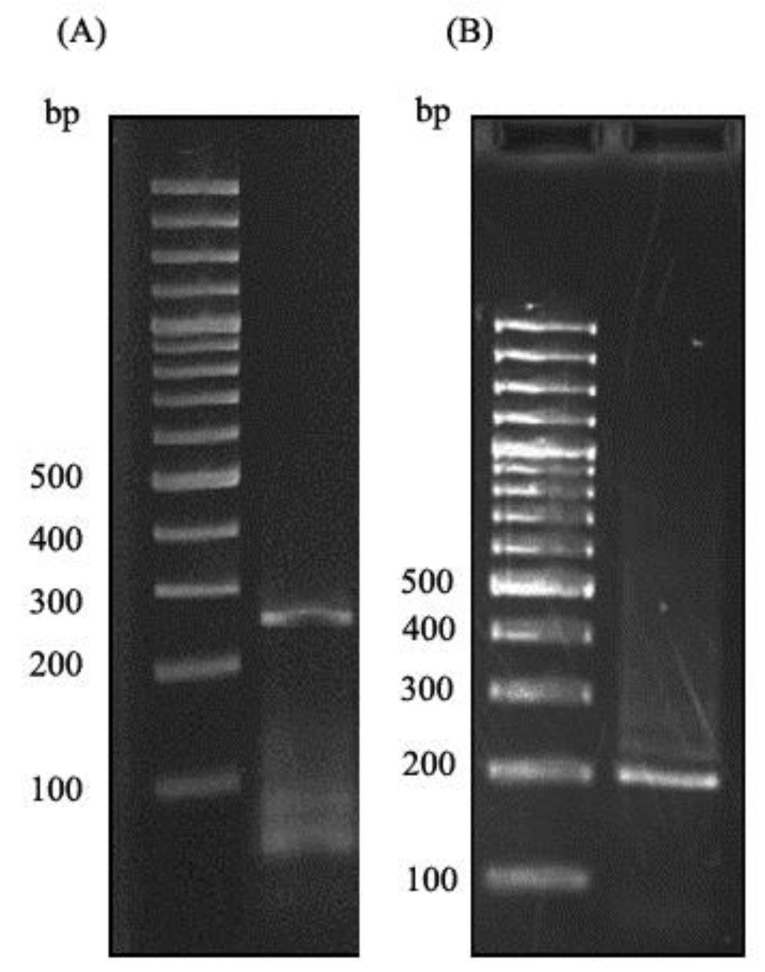
(A) The gel electrophoresis of PCR product with first set of primers using prepared cDNA library as the DNA template. (B) Gel electrophoresis of second PCR with second set of primers using the PCR product of first PCR as template.

**Figure 3:**
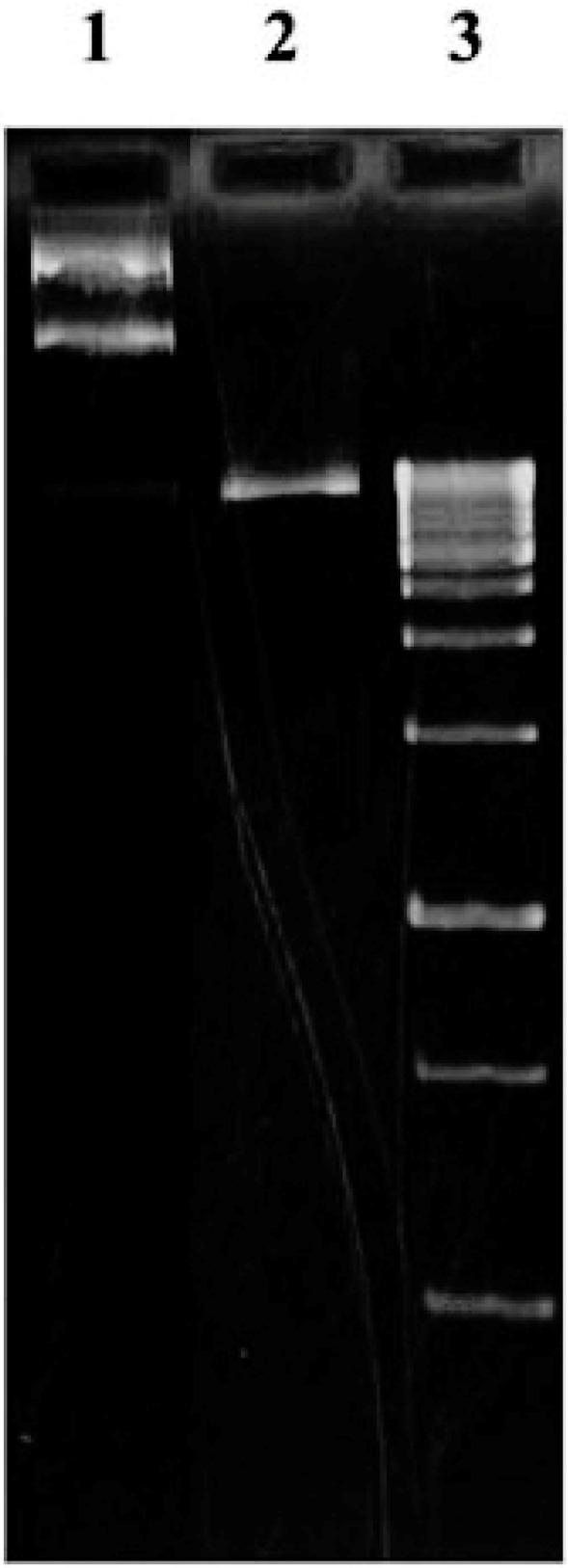
(1) Undigested pGEX-6P-1 supercoil plasmid, (2) the digested plasmid using BamHI and EcoRI enzymes, (3) DNA ladder.

**Figure 4:**
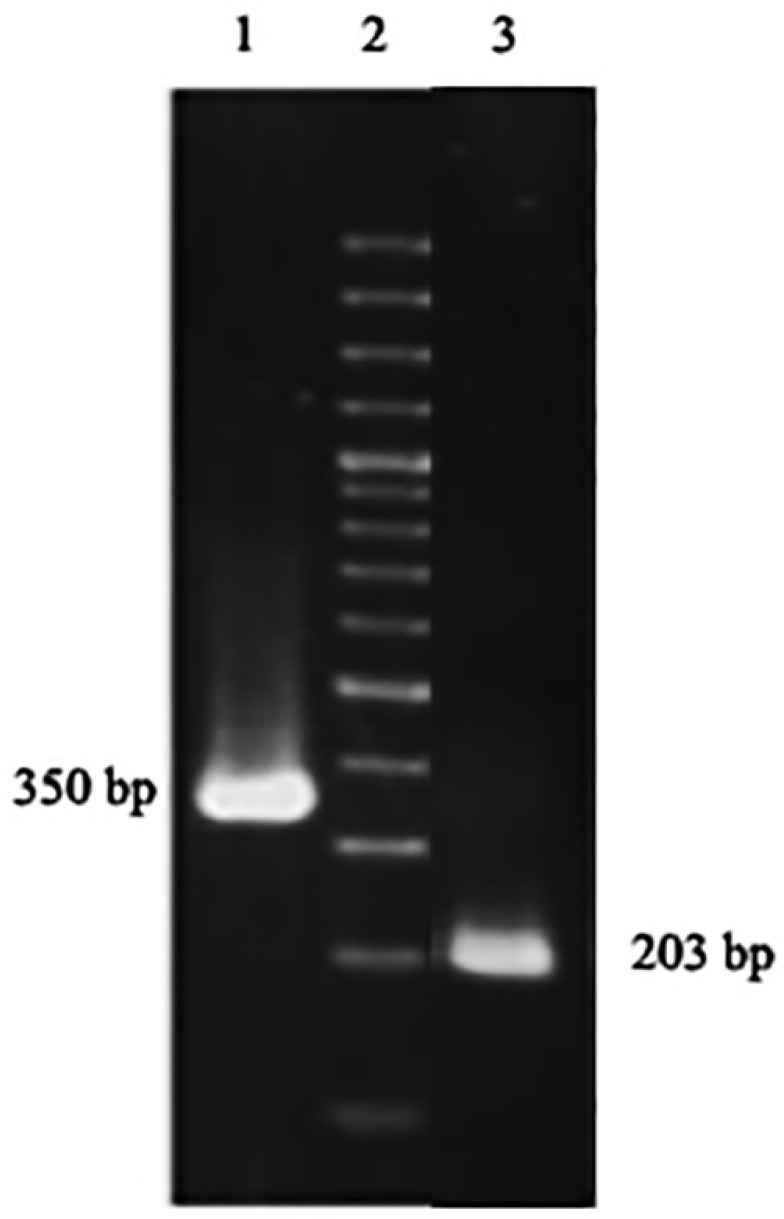
The gel electrophoresis of PCR products of DOF 4.2-ZF-pGEX-6P-1 construct using universal primers (lane 1), and F2 and R2 set of primers (lane 3).

**Twenty-day old***A. thaliana*plant was used to extract the total RNA. The extracted RNA was immediately converted to cDNA library, which then used in a nested PCR experiment to amplify the zinc finger domain of DOF4.2 (Figure 2). The product was digested using BamHI and EcoRI restriction enzymes and cloned into pGEX-6P-1 vector (Figure 3). The constructed vector was verified by PCR experiment targeting coding segment for DOF4.2 ZF prior to sequencing (Figure 4). DNA sequencing revealed that the DNA sequence of DOF 4.2-ZF is different in three positions compared to that of wild type *A. thaliana*species (Figure 1). Translation of the DNA sequence to corresponding protein showed that the first mutation is a missense resulting in serine to glycine mutation at position 18 of protein (Figure 1). The second mutation was silent (TAC→TAT) and did not lead to residue type change at position 19(Tyr). In the case of the third mutation, that was a nonsense mutation, the tryptophan codon was changed to stop codon at position 39 in DOF 4.2-ZF sequence. These mutations resulted in a DNA sequence encoding for truncated form of DOF 4.2-ZF (Figure 1).

### DOF4.2ZF domain expression

**Figure 5:**
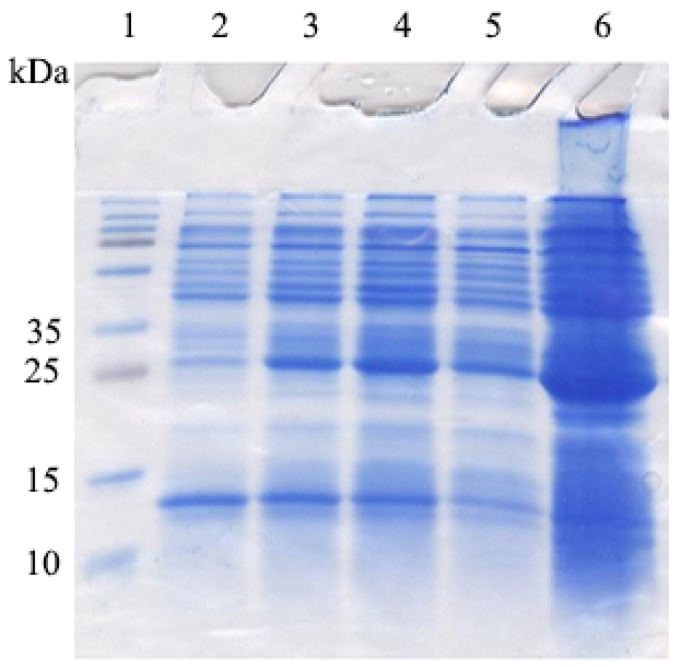
The SDS-PAGE gel electrophoresis of GST-DOF 4.2-ZF fusion protein. GST-DOF 4.2-ZF fusion protein with the size of 31 kDa can be observed. The lanes are samples prepared from soluble fraction of bacterial lysates: (2) before induction, (3) one hour, (4) two hours, (5) three hours after induction by 0.4 mM IPTG, (6) sample from the overnight culture.

**The DOF 4.2-ZF domain was expressed in***E. coli*BL21 strain. For this purpose, a single colony of *E. coli* BL21 containing pGEX-DOF 4.2-ZF was used to inoculate 10 mL of LB-ampicillin medium. After overnight incubation, the culture was diluted 1:100 in 100 mL LB-ampicillin and grown to OD of 0.6 before adding IPTG (0.4 mM). The samples taken before and after induction along with the overnight culture were analyzed on SDS-PAGE (Figure 5). The strong band with molecular weight at about 31 kDa stands for truncated DOF4.2-ZF fused to GST tag.

### In silico investigation of DOF 4.2-ZF–DNA interactions

**Figure 6:**
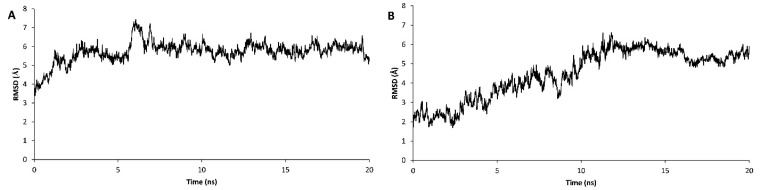
Plot of root mean square deviation (RMSD) fluctuation for the complex of DOF 4.2-ZF-DNA during 20 ns simulation period. Panels A and B show the results for the wild type and truncated form of DOF 4.2-ZF in complex with DNA, respectively.

**Figure 7:**
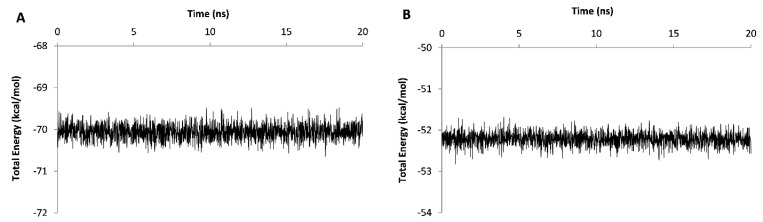
Plot of potential energy fluctuation for the complex of DOF 4.2-ZF-DNA during 20 ns molecular dynamics simulation period. Panels A and B show the results for the wild type and truncated form of DOF 4.2-ZF in complex with DNA, respectively.

**Figure 8:**
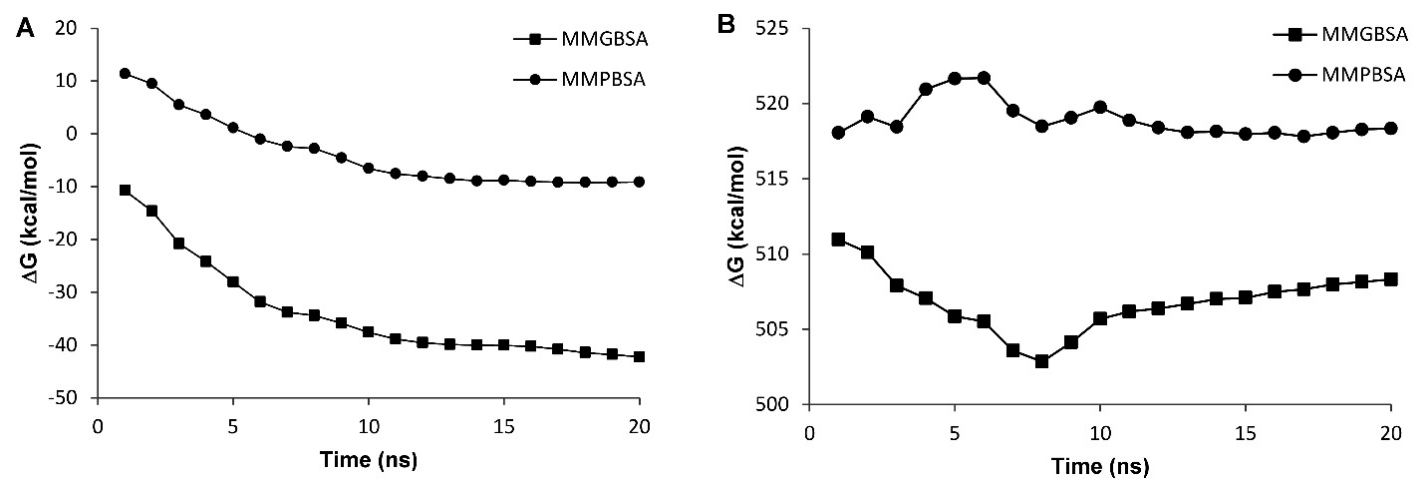
The plot of binding free energies (∆G) for the DOF 4.2-ZF in complex with DNA during different MD simulation time lengths using MM-PBSA/GBSA calculation methods implemented in AMBER. Panels A and B indicate the results of binding free energies for the wild type and truncated form of DOF 4.2-ZF in complex with DNA, respectively. The filled circles refer to MM-PBSA calculations while the filled squares demonstrate MM-GBSA calculations.

**In the current study, it was detected that the in house grown mutant variety of***A. thaliana*may express a truncated form of DOF 4.2 protein. Therefore, it was aimed to assess the DNA binding ability of this shortened form using molecular dynamics simulations. To this end, the 3D model of this truncated form was derived from and compared with the previously generated model for the wild type DOF 4.2-ZF built based on GATA1 protein.^26^ The stability assessment was carried out using MD simulations for 20 ns in which RSMD values were reached to a plateau after 4 and 13 ns for wild type and truncated DOF 4.2-ZF and remained steady for the rest of the simulations (Figure 6). Additional model validation was performed by analyzing the total energy of the systems which were steady during 20 ns MD simulations revealing the conformational stability of the models (Figure 7). In order to calculate the DNA binding energy for the truncated DOF 4.2-ZF and compare it to that of wild type, MM-GBSA and MM-PBSA algorithms implemented in AMBER suite were used. The results showed that truncated form of DOF 4.2-ZF may not interact with the corresponding DNA deduced from extremely positive value of the calculated binding free energy (Figure 8).

## Discussion

DOF proteins are a family of plant-specific transcription factors which contribute to numerous biological processes such as germination, dormancy, light and defense responses upon binding to DNA through a highly conserved DNA binding domain called DOF domain. In spite of great advances in the field of protein structure determination, efforts to produce and purify DOF domain proteins in order to solve their structures still remains a big challenge as these proteins tend to tightly bind to the corresponding DNA sequences. Taking all these facts into accounts, the current study aimed to produce and purify zinc finger domain of DOF 4.2 for biophysical studies with the aim of identify key interactions involved in DOF-DNA complexes. To do this, total RNA was extracted from *A. thaliana*plant and used for the synthesis of cDNA library. The gene of zinc finger domain of DOF 4.2 was amplified by a two-step PCR (nested PCR) strategy which can avoid nonspecific amplification of DNA segments related to ZF domains of other DOF proteins (Figure 2). After successful PCR amplification, the gene of interest was cloned at the C-terminus of GST protein using pGEX-6P-1 expression vector. The correctness of the constructed vector was assessed by PCR reactions (Figure 4) and subsequently DNA sequencing. Surprisingly, sequence analysis revealed that there are three nucleotide mutations in the DOF 4.2-ZF DNA sequence compared to the wild type (Figure 1). Translation of the obtained sequence showed that the mutation at position 19 is silent while the two others have resulted in missense mutation at the positions 18 (S18G) and nonsense mutation of tryptophan at position 39 to stop codon (Figure 1). Previous studies have shown the variation of amino acid sequence of DOF 4.2-ZF between the different members of DOF family in two conserved positions including C14S and R32C and the current study demonstrated new mutations in positions 18 and 39 of DOF ZF family, which the latter leads to a truncated form of DOF 4.2 protein. It seems that the aforementioned mutations did not have detrimental effects on germination and growth of the plant.

MD simulations have been extensively used to virtually investigate protein-protein/DNA and protein-small molecules interactions.^27-29^ Different studies have used MD simulation based algorithms to report the interaction of DOF family proteins with DNA.^26,30,31^ Pandey et al. explored the DNA binding ability of DOF domain from wheat showing that single K29R mutation could adversely affect its binding capability.^31^ In another study, we modeled different DOF zinc finger proteins and investigated their ability to bind DNA using MD simulations demonstrating that DOF 3.4 is able to bind DNA with more affinity compared to others.^26^ All these evidences show the applicability of MD simulations to calculate the binding energies of DOF protein to DNA in the absence of experimental data. The observation that the variants of *A. thaliana* used in the current study harbors mutations in the gene for DOF 4.2 protein not reported previously, prompted us to investigate the DNA binding properties of the resulting mutated protein using *in silico* methods. Analyzing the MD simulation results showed that the binding energies presented as ∆G_binding_ values were negative for the wild type indicating a favorable interaction. However, the values for the truncated ZF4.2 were positive (~500 kcal/mol) implying that the truncated form may not bind to DNA spontaneously (Figure 8).

## Conclusion

In the current study for the first time we report new variations in gene sequence of DOF 4.2 protein. These variations consist of three mutations in the DNA sequence of DOF 4.2-ZF domain from which one is silent, while the other two are missense and nonsense mutations. The result of these mutations is the production of truncated DOF 4.2 protein, which effectively means the elimination of almost three quarter of residues from its C-terminal. It is noteworthy that the mutant *A. thaliana* can still grow normally. In the current study, molecular dynamics simulation studies were carried out to compare the DNA binding ability of the produced truncated protein to that of the wild type. The results confirmed that the observed mutations are detrimental for the DNA binding ability of the truncated form of DOF4.2 which might be expressed in the mutant variant of *A. thaliana* used in this study.

## Ethical Issues

Not applicable.

## Conflict of Interest

The authors declare that this article content has no conflict of interest.

## Acknowledgments

The authors would like to thank the Research Office and Biotechnology Research Center of Tabriz University of Medical Sciences for providing financial support.
